# Comparative study between nalbuphine and dexmedetomidine for conscious sedation in patients undergoing colonoscopy: randomized comparative trial

**DOI:** 10.1186/s12871-025-03482-4

**Published:** 2025-11-28

**Authors:** Marwa M. Abdelrady, Hamdy A. Yousef, Khaled A. Khalaf, Omar A. Abulfadl, Mostafa H. Hassanien

**Affiliations:** 1 Anesthesia and Intensive Care Department, Faculty of Medicine, New Valley University, Kharga, Egypt; 2 Anesthesia and Intensive Care Department, Faculty of Medicine, Assiut University, Assiut, Egypt; 3 Department of Tropical Medicine and Gastroenterology, Faculty of Medicine, Assiut University, Assiut, Egypt

**Keywords:** Conscious sedation, Dexmedetomidine, Ramsay sedation scores, Elective colonoscopy, Nalbuphine

## Abstract

**Background:**

Sedation is increasingly preferred for patients undergoing colonoscopy to improve tolerance and procedural success. This study aimed to compare the sedative and analgesic profiles, as well as hemodynamic responses, of dexmedetomidine and nalbuphine during elective colonoscopies.

**Methods:**

A total of 66 patients scheduled for colonoscopy were randomly divided into two equal groups: one received an intravenous bolus of dexmedetomidine (1 µg/kg) followed by a continuous infusion (0.5 µg/kg/h), and the other received nalbuphine (0.2 mg/kg IV). The primary endpoint was the assessment of sedation depth, while secondary endpoints included pain perception, cardiovascular stability, recovery measures, satisfaction scores, and the occurrence of adverse effects.

**Results:**

Ramsay Sedation Scores (RSS) significantly differed between groups at 15, 20 min, and at the end of the procedure, with nalbuphine producing higher scores after 10 min. Recovery and discharge times were significantly longer in the nalbuphine group (*P* < 0.05). Both regimens were well tolerated, with no serious complications reported. Patient and endoscopist satisfaction were higher in the nalbuphine group (*P* < 0.05).

**Conclusion:**

Both dexmedetomidine and nalbuphine provided effective sedation during colonoscopy. Dexmedetomidine favored quicker recovery but was associated with greater bradycardia and hypotension. Nalbuphine, while inducing deeper sedation and superior post-procedural analgesia, was associated with longer recovery times but provided better hemodynamic stability.

**Trial registration:**

ClinicalTrials.gov (Identifier: NCT05689242), date 19/1/2023.

**Supplementary Information:**

The online version contains supplementary material available at 10.1186/s12871-025-03482-4.

## Introduction

Colonoscopy is the gold standard for diagnosing and screening colorectal diseases, significantly reducing cancer-related mortality through early detection and prevention [1, 2]. However, the procedure often causes discomfort due to air insufflation and endoscope manipulation, making sedation and analgesia essential to improve patient tolerance and procedural success.

The ideal sedative–analgesic regimen for colonoscopy should provide rapid onset, stable hemodynamics, swift recovery, and minimal respiratory or cardiovascular depression [3]. Agents such as propofol, midazolam, etomidate, dexmedetomidine, and opioids are commonly used, each with distinct pharmacological advantages and drawbacks [4]. Propofol remains widely favored for its fast onset and short duration, yet it lacks intrinsic analgesic properties and may cause dose-dependent cardiorespiratory depression or injection pain [5]. Combining propofol with opioids can reduce the propofol dose and improve safety, but opioid-related side effects such as nausea, vomiting, constipation, and delayed recovery remain problematic [6, 7].

Nalbuphine, a synthetic mixed κ-agonist and µ-antagonist opioid, provides potent analgesia with a ceiling effect on respiratory depression, making it a promising alternative for painless colonoscopy. It is associated with fewer opioid-related adverse effects, faster recovery, and better hemodynamic stability, features particularly suited for ambulatory endoscopy [8]. Despite these advantages, evidence regarding its sedative properties in procedural settings is limited [9].

Dexmedetomidine, a selective α₂-adrenergic receptor agonist, produces cooperative sedation and anxiolysis with minimal respiratory compromise [10, 11]. Its use allows patients to remain calm yet responsive, which facilitates smooth procedures without airway instrumentation.

Given the clinical need for safe, effective, and easily recoverable sedation strategies, this randomized comparative trial was conducted to evaluate the sedative, analgesic, and hemodynamic profiles of nalbuphine versus dexmedetomidine during elective colonoscopy.

## Patients and methods

This prospective, randomized, double-blind comparative clinical trial was conducted at Assiut University Hospital. Ethical approval was obtained from the Assiut Medical School Institutional Review Board (IRB No. 04–2023−200036), and the study was registered on ClinicalTrials.gov (Identifier: NCT05689242). The trial adhered to the ethical principles outlined in the Declaration of Helsinki and followed the CONSORT guidelines for reporting randomized controlled trials. Informed written consent was obtained from all participants before enrollment. The study duration spans from June 2023 to December 2024.

A total of 66 patients scheduled for elective colonoscopy under deep sedation (without the need for tracheal intubation) were recruited for the study. Eligible participants were aged between 45 and 65 years and categorized as American Society of Anesthesiologists (ASA) physical status class I or II. Inclusion required participants to provide informed consent and be accessible for telephone follow-up within four hours post-procedure. Exclusion criteria encompassed known hypersensitivity to dexmedetomidine or nalbuphine, prolonged opioid use, significant cardiac, hepatic, or renal impairment, pregnancy or breastfeeding, diagnosed obstructive sleep apnea or anticipated difficult airway, recent respiratory infection or asthma, a body mass index (BMI) >30 kg/m², or refusal to participate. The study protocol included predefined criteria for early termination, such as [1] a marked increase in adverse events [2], failure to achieve primary endpoints following interim analysis, or [3] a dropout rate exceeding 15%.

### Randomization and blinding

Randomization was performed using a computer-generated sequence to assign patients evenly (1:1 ratio) into two study groups. Allocation concealment was maintained using sequentially numbered, opaque, sealed envelopes. The 66 participants were therefore randomized into two equal groups (*n* = 33 per group). Double blinding was applied, with both the patients and outcome assessors unaware of group assignments. To ensure blinding integrity, the study medications were prepared and administered by an independent physician who was not involved in any other part of the trial. Additionally, the investigational drugs were placed in identical, opaque containers to further conceal the treatment allocation.

### Intervention protocol

- *Group I (Dexmedetomidine)*: Participants assigned to this group were administered an intravenous loading dose of dexmedetomidine at 1 µg/kg, diluted in 10 mL of normal saline and infused over 10 min. This was subsequently followed by a continuous infusion at a rate of 0.5 µg/kg/h, delivered using a 50-mL syringe via an electronic infusion pump.
- *Group II (Nalbuphine)*: Patients in this group received nalbuphine intravenously at a dose of 0.2 mg/kg, also diluted in 10 mL of normal saline and infused slowly over 10 min. Following the bolus, a placebo infusion of 50 mL normal saline was administered at an identical rate and duration using an electronic infusion pump to maintain blinding.

#### Procedural protocol

 All participants underwent standard pre-procedural preparation, including fasting and bowel cleansing using enemas. Upon arrival in the procedure room, baseline vital signs were recorded. Continuous physiological monitoring was initiated, comprising electrocardiography (ECG), non-invasive blood pressure measurement, pulse oximetry, and respiratory rate assessment. Supplemental oxygen was administered through a nasal cannula at a flow rate of 4 L/min. All procedures were performed by a senior consultant endoscopist with over 10 years of experience, supported by the same anesthesia team to minimize inter-operator variability.

Intravenous access was established with either an 18-G or 20-G cannula, and fluid therapy with Ringer’s lactate or normal saline was initiated based on each patient’s weight. Patients were positioned in the left lateral decubitus position with knees flexed. Emergency preparedness included having essential medications, oxygen delivery systems, endotracheal intubation tools, anesthesia machines, and other resuscitative equipment available. To ensure procedural consistency and reduce variability, all colonoscopies were performed by the same surgical team.

### Assessment parameters

#### Demographic and baseline data

Detailed demographic data were obtained for all participants, encompassing age, sex, body mass index (BMI), and classification according to the American Society of Anesthesiologists (ASA) physical status system.

#### Hemodynamic monitoring

Vital signs were monitored and documented at multiple time points: before administration of the study medication (baseline), immediately after drug infusion, at 5-minute intervals throughout the procedure, and during the first 30 min of the post-anesthesia care unit (PACU) stay. The recorded parameters included heart rate (HR), systolic blood pressure (SBP), diastolic blood pressure (DBP), mean arterial pressure (MAP), oxygen saturation (SpO₂), and respiratory rate. In addition, end-tidal carbon dioxide (EtCO₂) levels were continuously monitored using a nasal cannula.

#### Sedation assessment

Sedation levels were evaluated using the Ramsay Sedation Scale (RSS) [12]. At three predefined time points: immediately following the procedure, upon arrival in the post-anesthesia care unit (PACU), and 30 min after the procedure. The RSS consists of six points: 1: Fully awake (anxious, agitated, or restless), 2: Drowsy (cooperative, oriented, and tranquil), 3: Mild sedation (responds to commands only), 4: Moderate sedation (brisk response to light glabellar tap or loud auditory stimulus), 5: Deep sedation (sluggish response to stimuli), 6: Very deep sedation (no response to stimuli).

Throughout the colonoscopy, the target sedation level was an RSS score of 4 or higher. This threshold was used to ensure adequate sedation and minimize patient responses such as coughing, bucking, tearing, purposeful limb movement, or increases in heart rate and mean arterial pressure exceeding 20% from baseline values.

#### Time metrics

The following time metrics were recorded for each participant: induction time, colonoscopy duration, recovery time, and discharge time.

- *Induction time* was defined as the interval between the initiation of sedative drug administration and the commencement of the colonoscopy.
- *Colonoscopy time* refers to the period from endoscope insertion to its complete withdrawal.
- *Recovery time* was measured from the end of the procedure to the point at which the patient achieved an RSS score of 2, indicating light sedation with full responsiveness.
- *Discharge time* was defined as the duration from the end of the procedure to the patient reaching a Steward Recovery Score (SRS) of 6, indicating readiness for safe discharge [13].

Procedural success was determined based on two criteria: [1] successful completion of the colonoscopy, and [2] no need for additional or rescue sedation during the examination.

#### Pain assessment

Pain intensity was assessed using the Visual Analog Scale (VAS) at three distinct time points: immediately following the procedure, upon admission to the post-anesthesia care unit (PACU), and 30 min post-procedure. The VAS is a 10-point scale, where a score of 0 indicates the complete absence of pain and a score of 10 corresponds to the most severe pain imaginable.

#### Side effects

Perioperative adverse events were closely monitored and managed according to established clinical protocols. These included hypotension, hypertension, tachycardia, bradycardia, hypoxia, arrhythmias, dizziness, excessive secretions, bleeding, respiratory depression, nausea, and vomiting. The anesthesia team promptly addressed any deviations from normal physiological parameters.

For instance, hypoxia was treated by increasing the supplemental oxygen flow and initiating manual ventilation if oxygen saturation (SpO₂) dropped below 90%. Mild bradycardia and hypotension were initially managed by decreasing the infusion rate of the sedative agents and administering intravenous fluids, either crystalloids or colloids. Persistent bradycardia was treated with atropine 0.5 mg IV, repeated every 3–5 min as needed, up to a maximum dose of 3 mg. Hypotension unresponsive to fluid resuscitation was managed with intravenous vasopressors such as ephedrine (5–10 mg). Post-procedural nausea and vomiting were controlled using intravenous ondansetron at a dose of 4–8 mg.

- *Patients and surgeons’ satisfaction*: They rated their satisfaction at the end of the study period via a five-point Likert scale (1 - very dissatisfied, 2 - dissatisfied, 3 - neutral, 4 - satisfied, and 5 - very satisfied).

#### Outcomes

The primary endpoint of the study was to evaluate and compare the level of sedation achieved with nalbuphine versus dexmedetomidine, as measured by the Ramsay Sedation Scale (RSS). Secondary outcome measures included a comparative analysis of hemodynamic parameters, postoperative pain scores assessed via the Visual Analog Scale (VAS), recovery and discharge times, levels of satisfaction reported by both patients and endoscopists, and the incidence of perioperative adverse events across the two treatment groups.

#### Sample size

The sample size calculation was based on detecting differences in sedation scores, the primary outcome variable. A priori power analysis using t-tests for comparing two independent means indicated that a total of 62 participants (31 per group) would be adequate to achieve a statistical power of 75%, with a one-tailed alpha level of 0.05 and an effect size of 0.6. This calculation was performed using the G*Power software version 3.1.9.7. To account for anticipated dropouts, two additional participants were added to each group. Thus, a total of 66 patients were enrolled to ensure adequate power and data integrity.

#### Statistical analysis

Statistical analysis was conducted using SPSS version 25.0 (Statistical Package for the Social Sciences). The normality of data distribution was assessed through visual inspection of histograms and confirmed using the Shapiro-Wilk test. For variables following a normal distribution, comparisons between the two groups were performed using independent samples t-tests. In contrast, non-normally distributed data were analyzed using the Mann-Whitney U test for between-group comparisons and the Wilcoxon Signed Rank Test for within-group comparisons over time.

Categorical data were examined using Chi-square tests or Fisher’s Exact tests when appropriate. A p-value of less than 0.05 was considered statistically significant. Parametric results were reported as means ± standard deviations, while non-parametric data were summarized using medians and ranges. Categorical variables were presented as frequencies and percentages.

All randomized participants were analyzed according to their original group assignment. As no dropouts or missing data occurred, imputation was unnecessary.

## Results

**Fig. 1:**
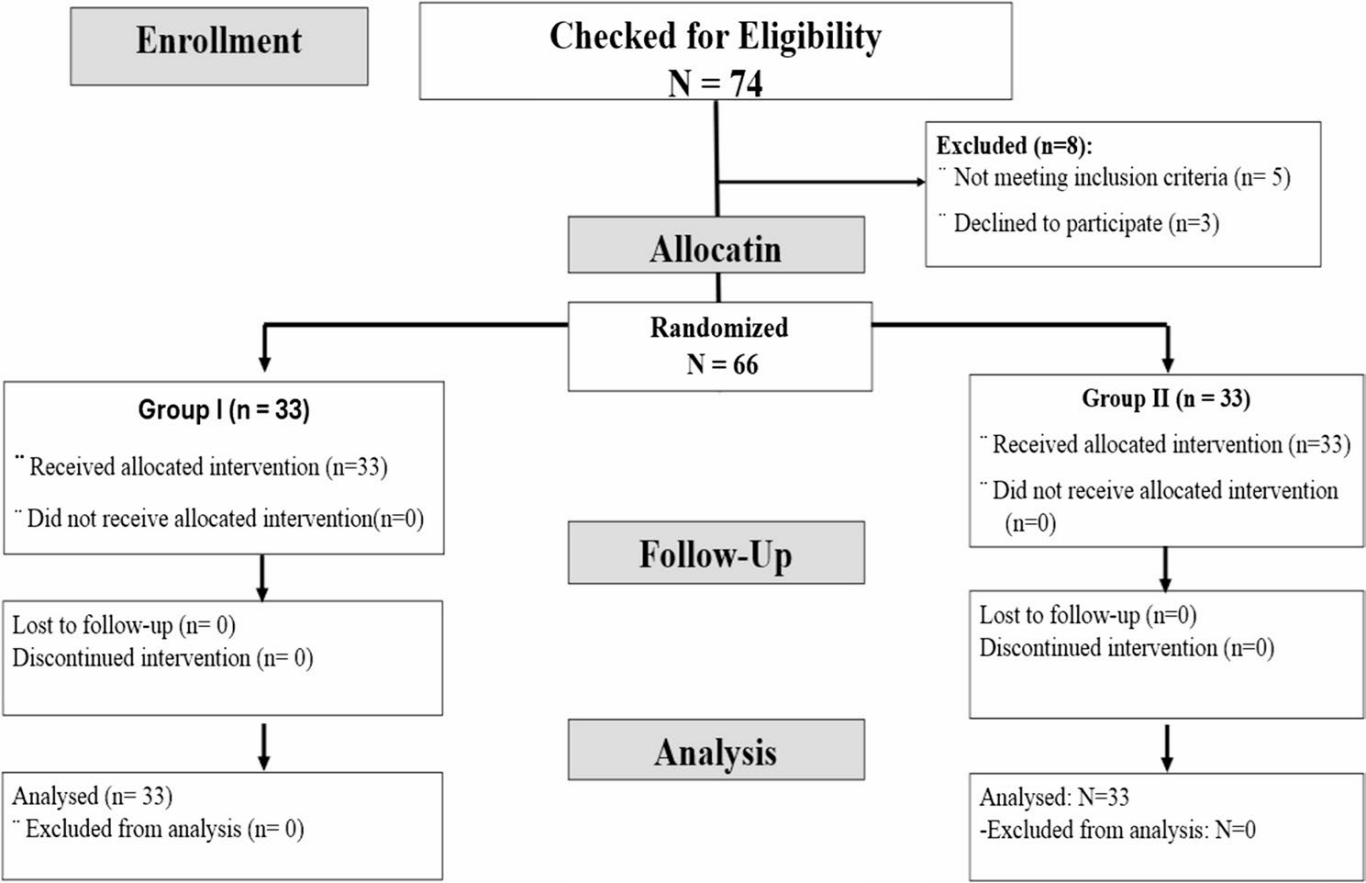
Consort flow chart

Figure 1 illustrates the CONSORT flow diagram detailing the process of patient enrollment, randomization, follow-up, and inclusion in the final analysis. Of the 74 patients initially assessed for eligibility, 8 were excluded, 3 declined to participate, and 5 did not meet the inclusion criteria. The remaining 66 participants were randomly assigned in a 1:1 ratio to either the dexmedetomidine or nalbuphine group (33 patients per group). All randomized patients completed the study protocol, and no additional exclusions occurred, resulting in a final analysis population of 66 participants (Fig. 1).

### Patient characteristics

No statistically significant differences were observed between the two groups concerning baseline characteristics, including age, body mass index (BMI), American Society of Anesthesiologists (ASA) classification, or gender distribution. However, patients in the nalbuphine group exhibited significantly longer induction, recovery, and discharge times compared to those in the dexmedetomidine group (*P* < 0.05). In contrast, the duration of the colonoscopy itself did not differ significantly between the groups (*P* > 0.05) (Table 1).

**Table 1:** Demographic data, induction time, colonoscopy time, recovery time, and discharge time between the Study Groups

	Group I Dexmedetomidine (*n* = 33)	Group II Nalbuphine (*n* = 33)	95% Confidence Interval of the Difference	*P* value
Age (years)	51.6 ± 5.3	51.9 ± 4.4	(−2.576- 2.027)	0.762
ASA I/II	25/8	23/10	---------	0.580
Sex F/M	15/18	19/14	---------	0.325
BMI	24.9 ± 5.2	24.6 ± 4.3	(−2.100- 2.585)	0.837
Induction time (sec)	10.7 ± 1.1	13.9 ± 1.1	(−3.699- −2604)	0.001*
Colonoscopy time (min)	11.8 ± 1.9	11.5 ± 1.7	(−0.540- 1.207)	0.448
Recovery time (min)	3.5 ± 0.5	3.9 ± 1.1	(−0.902- − 0.068)	0.023*
Discharge time (min)	19.3 ± 1.4	22.6 ± 2.7	(−4.352- −2.254)	0.001*

Continuous data were presented as Mean ± SD, Categorical data were presented as number (percentage) *p < 0.05, statistically significant differences

### Hemodynamics

There were no statistically significant differences between the two groups in terms of mean arterial pressure (MAP) and heart rate (HR) at any of the assessed time points (data not shown). The peripheral oxygen saturation SpO₂ remained ≥ 96% in all patients throughout the procedure, with no intergroup difference (*P* > 0.05)(Table 2). Both heart rate and non-invasive blood pressure (NIBP) remained stable throughout the entire procedure in both treatment groups.

#### Sedation

 Sedation was assessed using the Ramsay Sedation Scale (RSS) during the first 30 min following the procedure. There were no statistically significant differences in RSS values between the two groups from baseline up to 10 min post-induction. Both groups had a baseline RSS of 1, reflecting the absence of preoperative sedative administration. The target sedation level (RSS ≥ 4) was successfully achieved in the majority of patients in both groups following the initial induction dose.

However, from the 15-minute mark onward—including at 20 min and after the procedure, the nalbuphine group exhibited significantly higher RSS scores compared to the dexmedetomidine group (*P* < 0.05). Throughout the procedure, sedation levels in both groups remained within the range of 3 to 5, indicating adequate and consistent sedation. By the end of the procedure, sedation scores decreased to 2–3, with a median RSS of 2 in the dexmedetomidine group and 3 in the nalbuphine group. All patients in both groups reached an RSS of 1 at full recovery, which corresponded with achieving a Steward Recovery Score (SRS) of 6 (Table 3).

**Table 2:** SpO2 (%) in the two studied groups

SpO2 (%)		Dexmedetomidine Group (*N* = 33)	Nalbuphine Group (*N* = 33)	*p*-value
Baseline		98.90 ± 1.10	98.80 ± 1.00	0.712
After drug administration		98.80 ± 1.00	98.80 ± 1.00	0.892
After 5 min		98.70 ± 1.10	98.70 ± 1.10	0.945
After 10 min		98.60 ± 1.20	98.60 ± 1.10	0.876
After 15 min		98.50 ± 1.20	98.50 ± 1.20	0.923
After 20 min		98.40 ± 1.30	98.40 ± 1.20	0.867
After 25 min		98.30 ± 1.30	98.30 ± 1.20	0.892
After 30 min		98.20 ± 1.30	98.20 ± 1.20	0.934

*Abbreviations*: *SpO2* peripheral oxygen saturation, *SD* standard deviation

Continuous data are presented as Mean ± SD *p < 0.05 statistically significant differences

#### Postoperative VAS pain score

**Fig. 2:**
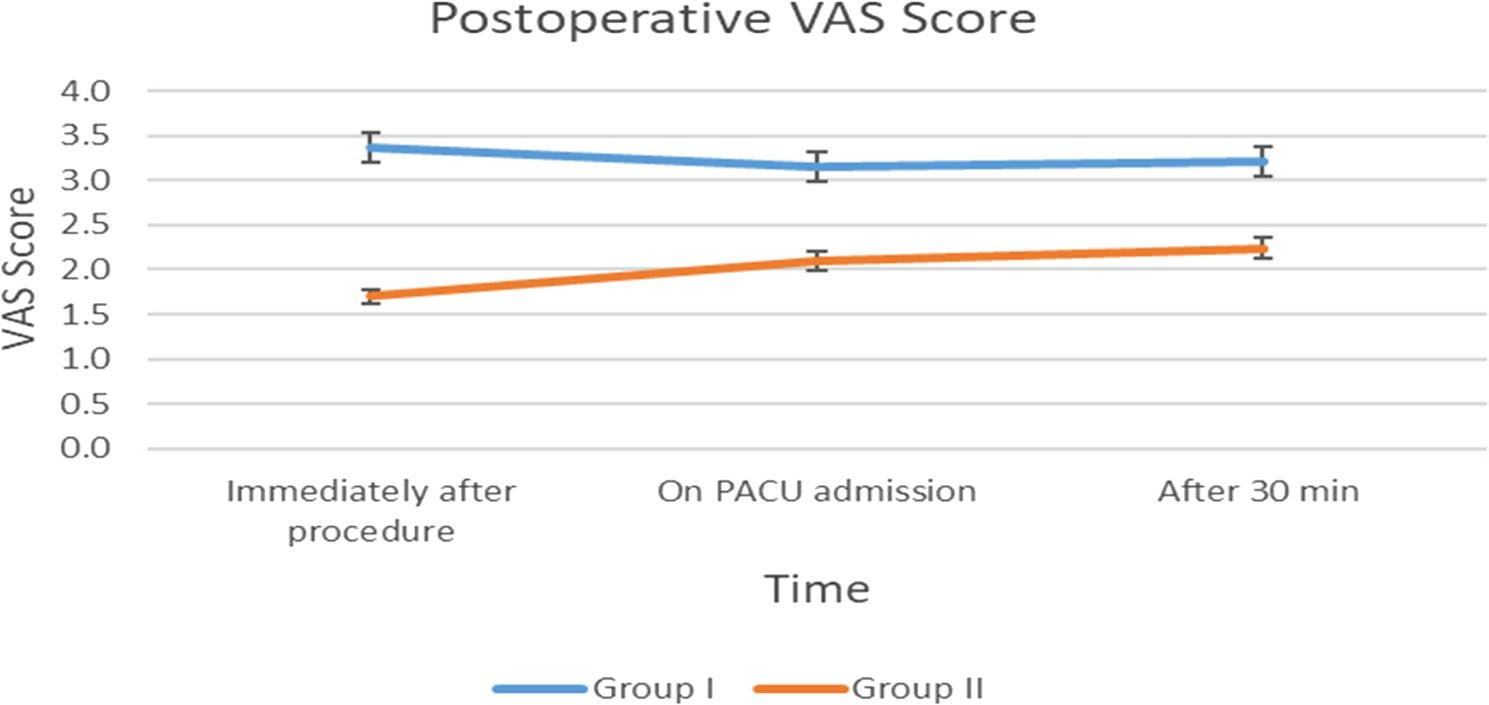
Visual Analog Scale (VAS) pain scores at different time points

 Postoperative pain was assessed using the Visual Analog Scale (VAS) at designated time intervals. Across all postoperative time points, patients in the nalbuphine group reported significantly lower VAS scores compared to those in the dexmedetomidine group (*P* < 0.05), indicating superior analgesic efficacy of nalbuphine in managing post-procedural discomfort. (Fig. 2).

#### Adverse events

 The most frequently observed adverse events in the post-anesthesia care unit (PACU) included bradycardia, hypotension, nausea, and vomiting. However, there were no statistically significant differences in the incidence of these side effects between the dexmedetomidine and nalbuphine groups (*P* > 0.05). Importantly, no severe or life-threatening adverse events were reported in either group during the study (Table 4).

**Table 3:** Ramsay sedation scores in between groups

	Group I (*n* = 33)	Group II (*n* = 33)	95% Confidence Interval of the Difference	*P* value
Baseline	1.5 ± 0.5	1.4 ± 0.5	(−0.188- 0.309)	0.627
Immediately after induction	2.9 ± 0.9	3 ± 0.8	(−0.501- 0.319)	0.659
5 min after induction	4.1 ± 0.8	4.15 ± 0.8	(−0.480- 0.298)	0.643
10 min after induction	4.12 ± 0.8	4.09 ± 0.8	(−0.379- 0.439)	0.883
15 min after induction	2.9 ± 0.8	4 ± 0.8	(−1.516- −0.727)	0.001*
20 min after induction	3.1 ± 0.8	4 ± 0.8	(−1.249- −0.448)	0.001*
At the end of the procedure	2.6 ± 0.5	3.5 ± 0.5	(−1.095- −0.602)	0.001*
RSS 30 min after procedure	1.8 ± 0.4	1.8 ± 0.8	(−0.320- 0.320)	1.000

Continuous data are presented as Mean ± SD. *p < 0.05 statistically significant differences

#### Surgeon and patients’ satisfaction

 Patient satisfaction was evaluated using a five-point Likert scale, with responses categorized as very satisfied, satisfied, neutral, dissatisfied, or very dissatisfied. A significantly higher proportion of patients in the nalbuphine group, approximately 94%, reported positive satisfaction (very satisfied, satisfied, or neutral), compared to 75.5% in the dexmedetomidine group (*P* < 0.05). Similarly, endoscopist satisfaction scores were also significantly higher among procedures performed under nalbuphine sedation (*P* < 0.05), suggesting greater procedural ease and patient cooperation (Tables 4).

**Table 4:** Postoperative side effects and Surgeon, patients’ Likert score

	Group I (*n* = 33)	Group II (*n* = 33)	*P* value
PONV	4(12.1%)	5(15.1%)	0.720
Bradycardia	3(9%)	3(9%)	1.000
Hypotension	3(9%)	4(12.1%)	0.689
Surgeon Satisfaction score			
Very satisfied	6(18.1%)	8(24.2%)	0.002*
Satisfied	16(48.5%)	8(24.2%)	
Neutral	4(12.1%)	16(48.5%)	
Dissatisfied	7(21.1%)	1(3%)	
Very dissatisfied	0	0	
Patient Satisfaction score			
Very satisfied	5(15%)	12(36.4%)	0.046*
Satisfied	9(27.2%)	5(15.1%)	
Neutral	11(33.3%)	14(42.4%)	
Dissatisfied	8(24.2%)	2(6%)	
Very dissatisfied	0	0	

Data presented number (%). Chi-square test

∗A statistically significant difference (*P* < 0.050)

∗∗A statistically significant difference (*P* < 0.001). PONV (Postoperative vomiting)

## Discussion

Deep sedation during colonoscopy has become the preferred approach in clinical practice, supported by multiple studies demonstrating its ability to enhance procedural success, patient comfort, and safety while facilitating faster recovery and minimizing hemodynamic instability [2]. The depth of sedation is a key determinant of procedural quality, directly influencing patient tolerance, procedural efficiency, and recovery dynamics.

Although opioids such as morphine and fentanyl are effective analgesics, their use in brief procedures is limited by adverse effects, controlled-substance regulations, and logistical constraints. Nalbuphine, by contrast, is inexpensive, readily available, and associated with a favorable safety profile, making it an attractive alternative for procedural sedation.

While both dexmedetomidine and nalbuphine are widely used in clinical practice, few studies have directly compared their sedative depth, hemodynamic stability, analgesic efficacy, recovery profile, and adverse event rates. Understanding these differences is crucial for optimizing sedation strategies in colonoscopy and selecting agents that balance comfort with safety.

In this randomized comparative trial, nalbuphine (0.2 mg/kg IV) produced significantly deeper sedation, superior post-procedural analgesia, and more stable hemodynamic parameters compared with dexmedetomidine, though with a modest prolongation in recovery time. Patient and endoscopist satisfaction were both higher in the nalbuphine group, without an increase in adverse events. The comparable rates of coughing, gagging, and hemodynamic fluctuations between groups confirm that nalbuphine maintained procedural safety while providing enhanced comfort.

Sedation achieved by both agents resembled “cooperative” or “arousable” sedation—patients remained calm yet responsive to verbal or tactile stimuli—like natural sleep. This contrasts with the deeper, less interactive sedation associated with GABAergic agents such as propofol or benzodiazepines [14]. Dexmedetomidine, primarily a sedative rather than a hypnotic, enables easy arousal and rapid recovery. Nasreen et al. also reported shorter awakening times with dexmedetomidine compared to placebo, supporting its role in facilitating quick post-procedure responsiveness [15].

In this study, Ramsay Sedation Scores (RSS) confirmed that nalbuphine produced significantly higher sedation levels beginning 10 min post-induction and persisting through the post-anesthesia period, likely due to κ-opioid receptor activation. Both agents provided effective procedural analgesia; however, nalbuphine demonstrated superior pain control, consistent with prior findings showing κ-receptor agonists to be particularly potent for visceral pain relief [16].

Respiratory depression is a common concern with opioids. Continuous monitoring of oxygen saturation and end-tidal CO₂ was used to detect early signs of hypoventilation. No significant difference in respiratory parameters was observed between groups, and neither produced clinically meaningful respiratory depression. This aligns with Gal et al., who showed that nalbuphine exhibits a ceiling effect on respiratory suppression [17], and with studies demonstrating a flatter dose–response curve for respiratory depression compared with morphine [18]. The dual action of nalbuphine—µ-receptor antagonism and κ-receptor agonism—limits respiratory compromise while preserving analgesia [19].

Dexmedetomidine’s main side effects are hemodynamic; however, the low-dose infusion used in this study effectively minimized clinically significant bradycardia or hypotension. Overall, both agents were well tolerated.

These findings support the hypothesis that nalbuphine’s analgesic efficacy, especially for visceral pain, may have been underestimated due to its mild side-effect profile. Its κ−2b receptor activity provides potent visceral analgesia, making it particularly suitable for gastrointestinal procedures such as colonoscopy. Moreover, its intrinsic sedative properties further enhance its clinical usefulness as a standalone or adjunct sedative-analgesic agent.

### Limitations

This study has several limitations that should be acknowledged. First, despite the use of conscious sedation, some patients may have retained awareness of the procedure. However, our findings suggest that this did not result in a significant increase in procedure-related discomfort. Second, although the power analysis indicated 75% statistical power—slightly below the conventional 80% threshold—this was accepted due to the limited number of eligible participants and resource constraints. Third, this may slightly reduce the study’s ability to detect smaller effect sizes. The observed differences in sedation depth between the two groups introduce a potential confounding factor; it remains unclear whether variations in respiratory depression were attributable to the inherent pharmacological properties of the drugs or simply to the level of sedation achieved. Fourth, consistent with prior studies, our results confirm that intravenous administration of dexmedetomidine is associated with a risk of bradycardia, even at clinically acceptable doses. This known side effect may limit its use in certain patient populations, particularly those with preexisting cardiac conduction abnormalities [20, 21]. Therefore, the use of dexmedetomidine for sedation should be approached with caution—or potentially avoided—in patients with baseline bradycardia or those receiving medications that influence cardiac conduction or heart rate. Additionally, fluid and electrolyte disturbances remain a relevant concern during bowel preparation for colonoscopy. Clinicians should be vigilant when administering dexmedetomidine in this context, as previous studies have indicated that the drug may exert a diuretic effect, potentially exacerbating water and electrolyte imbalances during the perioperative period [22, 23].Future research should aim to elucidate the impact of dexmedetomidine on fluid and electrolyte homeostasis during colonoscopy, particularly in the context of bowel preparation-induced shifts, to better define its safety profile and guide clinical practice. Lastly, the exclusion of obese patients (BMI >30 kg/m²) limits generalizability, since obesity is common among colonoscopy patients, and the relatively small sample size may limit the statistical power and generalizability of the findings.

## Conclusion

Both dexmedetomidine and nalbuphine proved to be effective agents for achieving conscious sedation during colonoscopy. Dexmedetomidine offered more consistent sedation levels and facilitated quicker recovery; however, it was associated with a higher incidence of bradycardia and hypotension. In contrast, nalbuphine produced deeper sedation, superior post-procedural analgesia, and more stable hemodynamic parameters, although recovery time was modestly extended. Therefore, the selection between these agents should be tailored to individual patient profiles, considering underlying comorbidities and the specific demands of the procedure. In clinical practice, nalbuphine may be preferred for patients where hemodynamic stability and post-procedural analgesia are priorities, whereas dexmedetomidine may be advantageous when rapid recovery is essential.

## Supplementary Information

Supplementary Material 1

## Data Availability

The datasets generated and/or analyzed during the current study are available from the corresponding author on reasonable request.

## Abbreviations

ASA: American Society of Anesthesiologists
BMI: Body Mass Index
CONSORT: Consolidated Standards of Reporting Trials
ECG: Electrocardiography
EtCO₂: End-Tidal Carbon Dioxide
F/M: Female/Male
HR: Heart Rate
MAP: Mean Arterial Pressure
NIBP: Non-Invasive Blood Pressure
PACU: Post-Anesthesia Care Unit
PONV: Postoperative Nausea and Vomiting
RSS: Ramsay Sedation Scale
SRS: Steward Recovery Score
VAS: Visual Analog Scale
IRB: Institutional Review Board

## References

[1] Deng C, Wang X, Zhu Q, Kang Y, Yang J, Wang H. Comparison of nalbuphine and sufentanil for colonoscopy: a randomized controlled trial. PLoS One. 2017;12(12):e0188901. 29232379 10.1371/journal.pone.0188901 PMC5726642

[2] Xu H, Tang RSY, Lam TYT, Zhao G, Lau JYW, Liu Y, et al. Artificial intelligence-assisted colonoscopy for colorectal cancer screening: a multicenter randomized controlled trial. Clin Gastroenterol Hepatol. 2023;21(2):337–e463. 35863686 10.1016/j.cgh.2022.07.006

[3] Wang X, Zhang M, Sun H, Zhang R, Zhu Y, Zhang Z, et al. Dexmedetomidine-oxycodone combination for conscious sedation during colonoscopy in obese patients: a randomized controlled trial. Heliyon. 2023;9(5):e16370. 37251861 10.1016/j.heliyon.2023.e16370 PMC10209023

[4] Childers RE, Williams JL, Sonnenberg A. Practice patterns of sedation for colonoscopy. Gastrointest Endosc. 2015;82(3):503–11. 25851159 10.1016/j.gie.2015.01.041 PMC4540687

[5] Chen HY, Deng F, Tang SH, Liu W, Yang H, Song JC. Effect of different doses of Dexmedetomidine on the median effective concentration of Propofol during Gastrointestinal endoscopy: a randomized controlled trial. Br J Clin Pharmacol. 2023;89(6):1799–808. 36527308 10.1111/bcp.15647

[6] Wang LL, Guan ZY, Wang CM, Zhang YW, Zhang J, Zhao P. A comparative study on the efficacy and safety of Propofol combined with different doses of alfentanil in gastroscopy: a randomized controlled trial. J Anesth. 2023;37(2):201–9. 36482231 10.1007/s00540-022-03145-5

[7] Seo B, Yang MS, Park SY, Park BY, Kim JH, Song WJ, et al. Incidence and economic burden of adverse drug reactions in hospitalization: a prospective study in Korea. J Korean Med Sci. 2023;38(8):e56. 36852852 10.3346/jkms.2023.38.e56 PMC9970790

[8] Hou C, Zhang S, Zhu Y, Wen G, Wang G, Dai J, et al. Comparative efficacy and safety of nalbuphine and hydromorphone in painless colonoscopy techniques: a randomized controlled trial. BMC Anesthesiol. 2025;25(1):187. 40240966 10.1186/s12871-025-03038-6 PMC12004782

[9] Chhabra A, Saini P, Sharma K, Chaudhary N, Singh A, Gupta S. Controlled hypotension for FESS: a randomised double-blinded comparison of magnesium sulphate and dexmedetomidine. Indian J Anaesth. 2020;64(1):24–30. 32001905 10.4103/ija.IJA_417_19 PMC6967369

[10] Zhao H, Davies R, Ma D. Potential therapeutic value of dexmedetomidine in COVID-19 patients admitted to ICU. Br J Anaesth. 2021;126(1):e33-5. 33678305 10.1016/j.bja.2020.09.031 PMC7531593

[11] Dere K, Sucullu I, Budak ET, Yeyen S, Filiz AI, Ozkan S, et al. A comparison of dexmedetomidine versus midazolam for sedation, pain and hemodynamic control during colonoscopy under conscious sedation. Eur J Anaesthesiol. 2010;27(7):648–52. 20531094 10.1097/EJA.0b013e3283347bfe

[12] Ramsay M, Savege T, Simpson B, Goodwin R. Controlled sedation with alphaxalone–alphadolone. Br Med J. 1974;2(5920):656–9. 4835444 10.1136/bmj.2.5920.656 PMC1613102

[13] Steward DJ. A simplified scoring system for the post-operative recovery room. Can Anaesth Soc J. 1975;22(1):111–3. 1109700 10.1007/BF03004827

[14] Yabäck-Karam V, Aouad MM. Perioperative uses of dexmedetomidine. Middle East J Anesthesiol. 2006;18(6):1043–56. 17263262

[15] Nasreen F, Bano S, Khan RM, Hasan SA. Dexmedetomidine used to provide hypotensive anesthesia during middle ear surgery. Indian J Otolaryngol Head Neck Surg. 2009;61(3):205–7. 23120636 10.1007/s12070-009-0067-8 PMC3449983

[16] Rivière PJ. Peripheral kappa-opioid agonists for visceral pain. Br J Pharmacol. 2004;141(8):1331–4. 15051626 10.1038/sj.bjp.0705763 PMC1574907

[17] Gal TJ, DiFazio CA, Moscicki J. Analgesic and respiratory depressant activity of nalbuphine: a comparison with morphine. Anesthesiology. 1982;57(5):367–74. 6814301 10.1097/00000542-198211000-00004

[18] Romagnoli A, Keats AS. Ceiling effect for respiratory depression by Nalbuphine. Clin Pharmacol Ther. 1980;27(4):478–85. 7357806 10.1038/clpt.1980.67

[19] Bailey PL, Clark NJ, Pace NL, Isern M, Stanley TH. Failure of nalbuphine to antagonize morphine: a double-blind comparison with naloxone. Anesth Analg. 1986;65(6):605–11. 3085551

[20] Lei H, Chao L, Miao T, Ya Jun L, Shen Ling L, Yan Ying P, et al. Incidence and risk factors of bradycardia in pediatric patients undergoing intranasal Dexmedetomidine sedation. Acta Anaesthesiol Scand. 2020;64(4):464–71. 31736052 10.1111/aas.13509

[21] Ahn EJ, Park JH, Kim HJ, Kim KW, Choi HR, Bang SR. Anticholinergic premedication to prevent bradycardia in combined spinal anesthesia and dexmedetomidine sedation: a randomized, double-blind, placebo-controlled study. J Clin Anesth. 2016;35:13–9. 27871510 10.1016/j.jclinane.2016.07.012

[22] Kirschen GW, Kim E, Adsumelli RSN. Dexmedetomidine-induced massive diuresis in a patient undergoing spinal fusion surgery: a case report and synthesis of the literature. A&A Pract. 2019;12(4):112–4. 10.1213/XAA.0000000000000860 30085933

[23] Ji F, Liu H. Intraoperative hypernatremia and polyuric syndrome induced by dexmedetomidine. J Anesth. 2013;27(4):599–603. 23377505 10.1007/s00540-013-1562-3

